# Improved growth rate in *Clostridium thermocellum* hydrogenase mutant via perturbed sulfur metabolism

**DOI:** 10.1186/s13068-016-0684-x

**Published:** 2017-01-03

**Authors:** Ranjita Biswas, Charlotte M. Wilson, Richard J. Giannone, Dawn M. Klingeman, Thomas Rydzak, Manesh B. Shah, Robert L. Hettich, Steven D. Brown, Adam M. Guss

**Affiliations:** 1 Biosciences Division, Oak Ridge National Laboratory, Oak Ridge, TN 37830 USA; 2 BioEnergy Science Center, Oak Ridge National Laboratory, Oak Ridge, TN 37830 USA; 3 Chemical Sciences Division, Oak Ridge National Laboratory, Oak Ridge, TN 37830 USA; 4 Centre for Rural Development and Technology, Indian Institute of Technology Delhi, Hauz Khas, New Delhi, 110016 India; 5 One Bethel Valley Road, Oak Ridge, TN 37831-6038 USA

**Keywords:** Cellulosic ethanol, *Clostridium thermocellum*, Redox balance, Metabolic engineering, Sulfate reduction

## Abstract

**Background:**

Metabolic engineering is a commonly used approach to develop organisms for an industrial function, but engineering aimed at improving one phenotype can negatively impact other phenotypes. This lack of robustness can prove problematic. Cellulolytic bacterium *Clostridium thermocellum* is able to rapidly ferment cellulose to ethanol and other products. Recently, genes involved in H_2_ production, including the hydrogenase maturase *hydG* and NiFe hydrogenase *ech*, were deleted from the chromosome of *C. thermocellum*. While ethanol yield increased, the growth rate of Δ*hydG* decreased substantially compared to wild type.

**Results:**

Addition of 5 mM acetate to the growth medium improved the growth rate in *C. thermocellum ∆hydG*, whereas wild type remained unaffected. Transcriptomic analysis of the wild type showed essentially no response to the addition of acetate. However, in *C. thermocellum ΔhydG*, 204 and 56 genes were significantly differentially regulated relative to wild type in the absence and presence of acetate, respectively. Genes, Clo1313_0108-0125, which are predicted to encode a sulfate transport system and sulfate assimilatory pathway, were drastically upregulated in *C. thermocellum ΔhydG* in the presence of added acetate. A similar pattern was seen with proteomics. Further physiological characterization demonstrated an increase in sulfide synthesis and elimination of cysteine consumption in *C. thermocellum ΔhydG*. *Clostridium thermocellum ΔhydGΔech* had a higher growth rate than *ΔhydG* in the absence of added acetate, and a similar but less pronounced transcriptional and physiological effect was seen in this strain upon addition of acetate.

**Conclusions:**

Sulfur metabolism is perturbed in *C. thermocellum ΔhydG* strains, likely to increase flux through sulfate reduction to act either as an electron sink to balance redox reactions or to offset an unknown deficiency in sulfur assimilation.

**Electronic supplementary material:**

The online version of this article (doi:10.1186/s13068-016-0684-x) contains supplementary material, which is available to authorized users.

## Background

Microbial conversion of lignocellulosic biomass to fuels, chemicals, and products holds promise as a sustainable approach to replacing ones derived from petroleum. While no known organism is capable of producing cellulosic biofuels at high yield and titer, metabolic engineering has the potential to create biocatalysts capable of economic production of biofuels. However, engineered organisms often have growth defects, and this lack of robustness would prevent industrial deployment.

*Clostridium thermocellum* is a thermophilic, cellulolytic bacterium that has the potential to perform one-step hydrolysis and fermentation of plant biomass without added enzymes by a process called consolidated bioprocessing (CBP) [1]. However, wild-type *C. thermocellum* is limited by its low ethanol yield and titer, producing acetate, lactate, H_2_, formate, free amino acids, and other compounds as additional fermentation products [2, 3]. Recent development of genetic tools for *C. thermocellum* [4–9] has enabled the construction of numerous targeted mutants, eliminating acetate, lactate, formate, and H_2_ production [4, 7, 10–14]. One of the mutations, *C. thermocellum ΔhydG*, eliminated a hydrogenase maturase protein involved in assembly of the [FeFe]-active site of three of the four hydrogenases in *C. thermocellum*. This deletion resulted in decreased H_2_ production and increased ethanol yield, but also a diminished growth rate. Further deletion of the [NiFe] hydrogenase Ech completely abolished H_2_ production [11]. Because blocking H_2_ production alters electron flux, it is possible that this mutation causes a redox imbalance, which could also explain the slower growth. Further supporting this hypothesis, the *∆hydG* mutant acquired a point mutation in the bifunctional aldehyde/alcohol dehydrogenase (*adhE*). This mutation expanded the substrate specificity to include not only NADH as an electron donor, but also NADPH [11], which could partially alleviate redox problems by giving the cell more pathways to balance redox reactions.

Understanding additional mechanisms used by *C. thermocellum* to balance redox reactions will be important for future metabolic engineering efforts. Here, we found that the addition of acetate to the culture medium increased the growth rate of *C. thermocellum ΔhydG*. We, therefore, utilized a combination of transcriptomics, proteomics, and physiological characterization to better understand this phenomenon.

## Methods

### Strains and culture conditions

*Clostridium thermocellum* DSM1313 and mutant strains *C. thermocellum Δhpt ΔhydG* (referred to as *ΔhydG*) and *C. thermocellum Δhpt ΔhydG Δech* (referred to as *ΔhydG Δech*) [11] were grown in CTFUD medium [6] and MTC minimal medium [15] prepared as described in [16]. CTFUD medium composition was (L^−1^): 3 g sodium citrate tribasic dehydrate, 1.3 g ammonium sulfate, 1.43 g potassium phosphate monobasic, 1.8 g potassium phosphate dibasic trihydrate, 0.5 g cysteine-HCl, 10.5 g 3-morpholino-propane-1-sulfonic acid (MOPS), 6 g glycerol-2-phosphate disodium, 5 g cellobiose, 4.5 g yeast extract, 0.13 g calcium chloride dehydrate, 2.6 g magnesium chloride hexahydrate, 0.0011 g ferrous sulfate heptahydrate, and 0.0001 g resazurin, adjusted to pH 7.0. MTC medium consisted of (L^−1^): 2 g sodium citrate dehydrate, 1.25 g citric acid monohydrate, 1 g sodium sulfate, 1 g potassium phosphate dibasic trihydrate, 2.5 g sodium bicarbonate, 1.5 g ammonium chloride, 2 g urea, 1 g magnesium chloride hexahydrate, 0.2 g calcium chloride dehydrate, 0.1 g ferrous chloride tetrahydrate, 1 g l-cysteine hydrochloride monohydrate, 5 g cellobiose, 0.001 g resazurin, 5 g MOPS, 20 mg pyridoxamine dihydrochloride, 1 mg riboflavin, 1 mg nicotinamide, 0.5 mg DL-thioctic acid, 4 mg 4-amino benzoic acid, 4 mg D-biotin, 0.025 mg folic acid, 2 mg cyanocobalamin, 4 mg thiamine hydrochloride, 0.5 mg MnCl_2_·4H_2_O, 0.5 mg CoCl_2_·6H_2_O, 0.2 mg ZnSO_4_·7H_2_O, 0.05 mg CuSO_4_·5H_2_O, 0.05 mg HBO_3_, 0.05 mg Na_2_MoO_4_·2H_2_O, and 0.05 mg NiCl_2_·6H_2_O.

### Whole-genome resequencing

Genome resequencing was performed by the Department of Energy Joint Genome Institute (JGI, Walnut Creek, CA) using an Illumina MiSeq instrument. Genomic DNA was extracted using a Qiagen DNeasy kit (Qiagen, Valencia, CA), was sheared to 500 bp fragments using the Covaris LE220 ultrasonicator (Covaris), and size selected using AMPure XP SPRI beads (Beckman Coulter). The fragments were treated with end-repair, A-tailing, and ligation of Illumina compatible adapters (IDT, Inc) using the KAPA-Illumina library creation kit (KAPA Biosystems). The prepared libraries were quantified using KAPA Biosystem’s next-generation sequencing library qPCR kit and run on a Roche LightCycler 480 real-time PCR instrument. The quantified multiplexed libraries were pooled in sets of 10, and sequenced on the Illumina MiSeq sequencer using an indexed PE150 protocol with MiSeq V2 chemistry.

Resequencing data analysis was performed using QIAGEN Bioinformatics CLC Genomics Workbench (http://www.qiagenbioinformatics.com/products/clc-genomics-workbench), which incorporates a comprehensive set of analysis tools for Next-Generation Sequencing data. Paired-end reads were mapped to the reference genome [Genbank: CP002416] using the built-in Map Reads to Reference Tool. Further refinement of the reads mapping was performed by the Local Realignment Tool, which attempts to re-align each mapped read by exploiting the alignment information of *other* mapped reads. Realignment typically occurs in areas around insertions and deletions in the sample reads relative to the reference, resulting in more accurate mapping. Mapped reads were next analyzed by the built-in tools Basic Variant Detection Tool for putative SNV and MNV detection, and InDels and Structural Variants Tool for detection of putative structural variants. Variants occurring in <90% of the reads and variants that were identical to those of the parent *Δhpt* strain (e.g., due to errors in the reference sequence or mutations present at the beginning of strain construction) were filtered out. Raw data are available from the JGI Sequence Read Archive (JGI Project Id: 1053867 and 1053888).

### Fermentation conditions

The inoculum for batch fermentation was prepared by growing the mutants in MTC medium overnight at 55 °C in an anaerobic chamber (COY Laboratory Products, Grass Lake, MI). The fermentation was carbon limited and carried out in 27 mL Balch tubes with 10 mL of MTC medium containing 5 g L^−1^ of cellobiose as the carbon source, supplemented with 5 mM sodium acetate where noted, under a N_2_ headspace sealed with butyl rubber stoppers. The tubes were inoculated with 0.5% v/v culture and incubated at 55 °C. The fermentation products were determined after 53 h of growth. Final cellobiose concentration was usually <0.5 mM, suggesting that fermentation activity was complete. Fermentations were performed at least two times with three independent biological replicates each. The “No Acetate” data were previously reported [11], which were generated simultaneously with the “Added Acetate” data reported here.

### Analytical methods

Fermentation products, including ethanol, acetate, lactate, and formate, were analyzed on Breeze 2 High-Performance Liquid Chromatograph system (Waters Corp, Milford, MA) using an Aminex-HPX-87H column with a 5 mM sulfuric acid mobile phase. Sulfide was measured using an Orion silver ion selective electrode (Thermo Fisher Scientific, Waltham, MA) as previously described [17]. H_2_ was measured using a 6850 Series II Gas Chromatograph (Agilent Technologies, Santa Clara, CA) using a thermal conductivity detector at 190 °C with a N_2_ reference flow and a Carboxen 1010 PLOT (30.0 m × 530 µm I.D.; model Supelco 25467) column.

### RNA isolation

The cells were grown to an OD of 0.3–0.4 in CTFUD medium, centrifuged at 4 °C for 5 min, and immediately flash frozen in liquid N_2_. Pelleted cells were resuspended in 1.5 mL of TRIzol (Invitrogen, Carlsbad, CA). Glass beads (0.8 g of 0.1 mm glass beads; BioSpec Products, Bartlesville, OK) were added to the cell suspension and lysed with 3 × 20 s bead beating treatments at 6500 rpm in a Precellys 24 high-throughput tissue homogenizer (Bertin Technologies, Montigny-le-Bretonneux, France). Total RNA was purified using an RNeasy kit (Qiagen, Valencia, CA) with DNase I on-column treatment. RNA quantity was determined by NanoDrop ND-1000 spectrophotometer (Thermo Fisher Scientific) and RNA quality was assessed with Agilent Bioanalyzer (Agilent Technologies). RNA (10 µg) was used as the template to generate ds-cDNA using Invitrogen ds-cDNA synthesis kit according to the manufacturer’s protocols (Invitrogen).

### Microarray sample labeling, hybridization, scan, and statistical analysis of array data

The ds-cDNA was labeled, hybridized, and washed according to the NimbleGen protocols. Hybridizations were conducted using a 12-bay hybridization station (BioMicro Systems, Salt Lake City, UT) and the arrays dried using a Maui wash system (BioMicro Systems). Microarrays were scanned with a Surescan high-resolution DNA microarray scanner (5 µm) (Agilent Technologies), and the images were quantified using the NimbleScan software (Roche NimbleGen, Madison, WI). Raw data were log2 transformed and imported into the statistical analysis software JMP Genomics 6.0 (SAS Institute, Cary, NC). The data were normalized together using a single round of the LOESS normalization algorithm within JMP Genomics, and distribution analyses were conducted before and after normalization as a quality control step. An ANOVA was performed in JMP Genomics to determine differential expression levels between conditions using the False Discovery Rate (FDR) testing method (p < 0.05). Microarray data have been deposited in NCBI Gene Expression Omnibus (GEO) database under accession number (GSE54082). Data are average of three independent biological replicates.

### Real-time quantitative-PCR (RT-qPCR) analysis

Microarray data were validated using real-time qPCR, as described previously [18]. Based on microarray hybridizations of *C. thermocellum* mutants, a set of 5 genes (Clo1313_0115, Clo1313_0147, Clo1313_0372, Clo1313_1559, and Clo1313_2243) representing a range of gene expression values was analyzed using qPCR from cDNA prepared for microarrays. Oligonucleotide sequences of the primers targeting the five genes selected for qPCR analysis are shown in Additional file 1: Table S1. Data are average of three independent biological replicates.

### Proteomics analysis

Crude protein fraction of *C. thermocellum* cell pellet was processed and digested with trypsin, and peptides were eluted and analyzed over an 11-step MudPIT as described previously [19]. High mass accuracy was utilized for both MS1 (30 K resolution) and MS2 (7.5 K resolution; CID) scans (1 microscan each), with data-dependent acquisition settings as follows: 1 full scan followed by 20 MS/MS scans, isolation window = 2.1 m/z, dynamic exclusion window, duration, and max = −0.52/+ 1.02 m/z, 15 s, and 500, respectively. Peptides generated from *C. thermocellum* strain DSM1313 FASTA database concatenated with common contaminants and reversed entries were matched to MS/MS spectra using MyriMatch v. 2.1 [20]. Common sample prep-induced modifications, i.e., Cys + 57.0214 Da (alkylation; static), Met + 15.9949 Da (oxidation; dynamic), and N-terminus + 43.0058 Da (carbamylation; dynamic), were included in the search parameters. Matches were filtered and assembled using IDPicker v. 3.0 [21] using a minimum of two distinct peptides per protein identification and adjusting the minimum spectra count (SpC) per protein to achieve protein level FDRs < 5%, peptide-level FDRs < 1%, and PSM-level FDRs < 0.25%. Protein identifications with associated spectral counts (SpC) were tabulated, balanced, and normalized for semi-quantitative proteomics as previously described [18]. Normalized SpC (nSpC) were used as a proxy for protein abundance across individual samples. To assess differences in protein abundance, the top 99% of total assigned spectra (across all sample conditions) was log2-transformed and processed by ANOVA (JMP Genomics v. 4.1) to assess statistical significance. Proteins with significant differences in abundance (*p* value ≤0.01) and minimum of twofold change were identified and compared with transcriptomics data to identify proteins affected by the knock-out of hydrogenases as well as the addition of acetate to the culture. Data are average of three independent biological replicates. The mass spectrometry proteomics data have been deposited to the ProteomeXchange Consortium (http://www.proteomexchange.org) via the PRIDE partner repository with the data set identifier PXD000777.

## Results

### Addition of acetate increased growth rate of *ΔhydG* strains

**Fig. 1:**
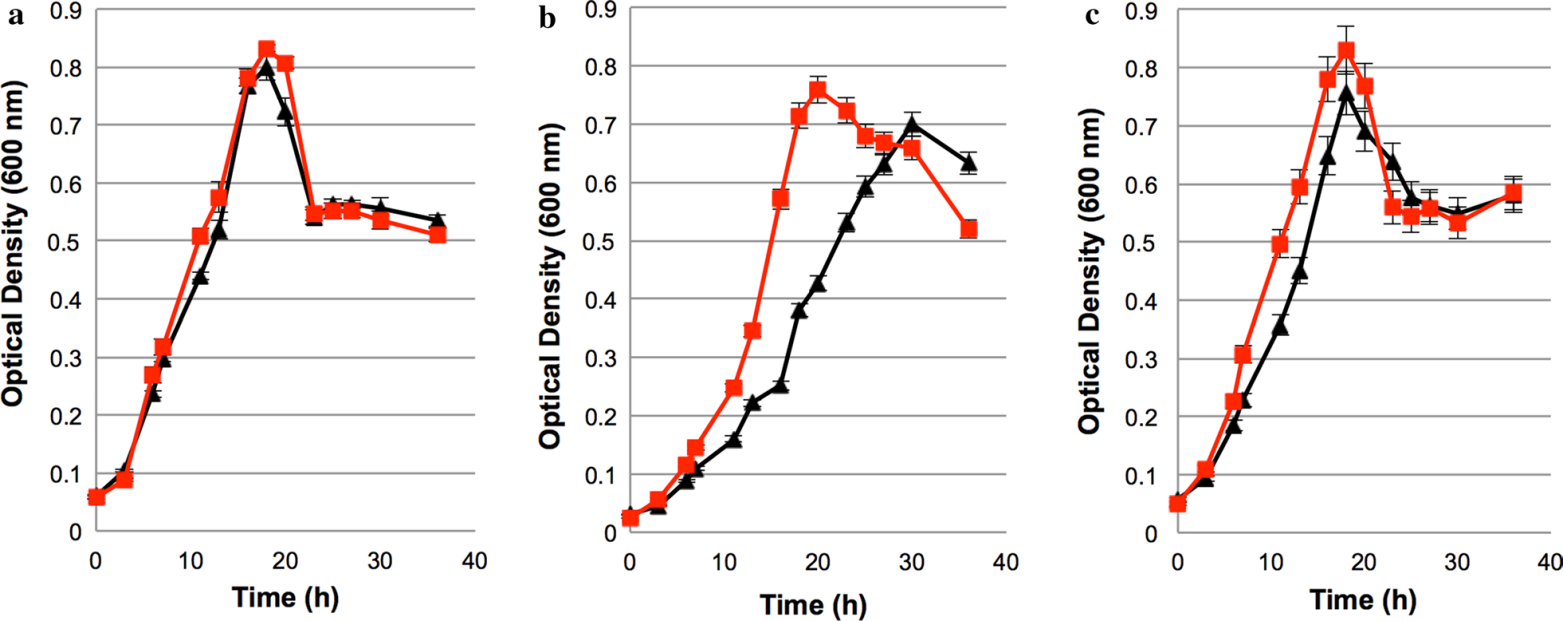
Growth profile of *C. thermocellum* strains on minimal medium. **a** Wild type, **b** *ΔhydG*, and **c** *ΔhydG Δech*. *Symbols*: *red square*, with added acetate; *black triangle*, without added acetate. Data for “without added acetate” are from [11]

Over the course of strain construction, we observed that growth of the *C. thermocellum ΔhydG* mutant was improved by the addition of exogenous acetate. Acetate was initially added based on the hypothesis that the addition of acetate could decrease flux through the acetate formation pathway [22], which could help prevent redox imbalances in the absence of H_2_ production. Indeed, acetate was a component of the culture medium during *C. thermocellum ΔhydG Δech* strain construction, and we were only successful in deleting *ech* in *C. thermocellum ΔhydG* when the medium contained added acetate. To characterize this growth phenotype, wild type, *ΔhydG* and *ΔhydG Δech* were grown in medium supplemented with 5 mM acetate. Growth of the wild-type strain was unaffected by the addition of acetate (Fig. 1a; 0.26 and 0.3 h^−1^, respectively, with nearly perfectly overlapping growth curves). However, in the presence of added acetate, the *∆hydG* strain grew 33% more rapidly (0.16 h^−1^) than without added acetate (0.12 h^−1^) and attained a maximum optical density of 0.76 within 20 h (Fig. 1b), as opposed to 30 h in the absence of added acetate. While less pronounced, addition of acetate to the growth medium increased the growth rate of *∆hydG ∆ech* as well, from 0.22 to 0.24 h^−1^ (Fig. 1c). While the growth rate was improved, the fermentation product profile did not substantially change upon addition of acetate (Table 1), with an ethanol yield similar to that reported previously without added acetate [11]. Individual amino-acid concentrations are presented in Additional file 2: Figure S2 and are not substantially different from [11].

**Table 1:** Fermentation product profile of *C. thermocellum* strains in absence/presence of added acetate to minimal medium

	Wild type		*ΔhydG*		*ΔhydGΔech*	
	No acetate^c^	Added acetate	No acetate^c^	Added acetate	No acetate^c^	Added acetate
Ethanol^a^	19.63 ± 2.98	22.88 ± 1.82	30.94 ± 1.61	31.43 ± 0.6	35.84 ± 1.0	36.06 ± 1.62
Acetate^b^	9.50 ± 1.9	8.90 ± 1.50	2.56 ± 0.07	1.10 ± 0.6	3.46 ± 0.36	1.76 ± 0.90
Lactate	3.84 ± 1.03	3.52 ± 1.56	0.11 ± 0.15	0.07 ± 0.2	0.12 ± 0.50	0.1 ± 0.98
Formate	6.54 ± 0.82	5.73 ± 1.17	4.33 ± 0.01	4.0 ± 0.01	6.8 ± 0.04	3.84 ± 0.03
Amino acids	4.1 ± 0.24	4.5 ± 0.09	2.83 ± 0.10	3.41 ± 0.04	2.14 ± 0.08	2.93 ± 0.13
Hydrogen	14.53 ± 3.36	14.04 ± 3.14	1.17 ± 0.37	0.96 ± 0.42	ND	ND

*ND* not detected

^a^All values are reported in mmol/L

^b^Net acetate production

^c^Data previously reported [11]

To balance redox reactions, each molecule of synthesized acetate requires the concomitant production of two molecules of a more reduced compound (e.g., formate or H_2_). Furthermore, there is a net production of NAD(P)H during microbial biomass formation from sugars [23]. Thus, one would expect more formate and H_2_ to be produced than would be needed to balance the production of acetate alone. In wild type, 2 mM extra (formate + H_2_) was synthesized relative to the amount that would be required to balance acetate production (Additional file 1: Table S2). The mutant strains, on the other hand, produced approximately zero additional (formate + H_2_) beyond what is needed to balance acetate production, raising the possibility that an additional electron sink could be in use.

### Sulfate metabolism genes are upregulated in *∆hydG* with addition of acetate

**Fig. 2:**
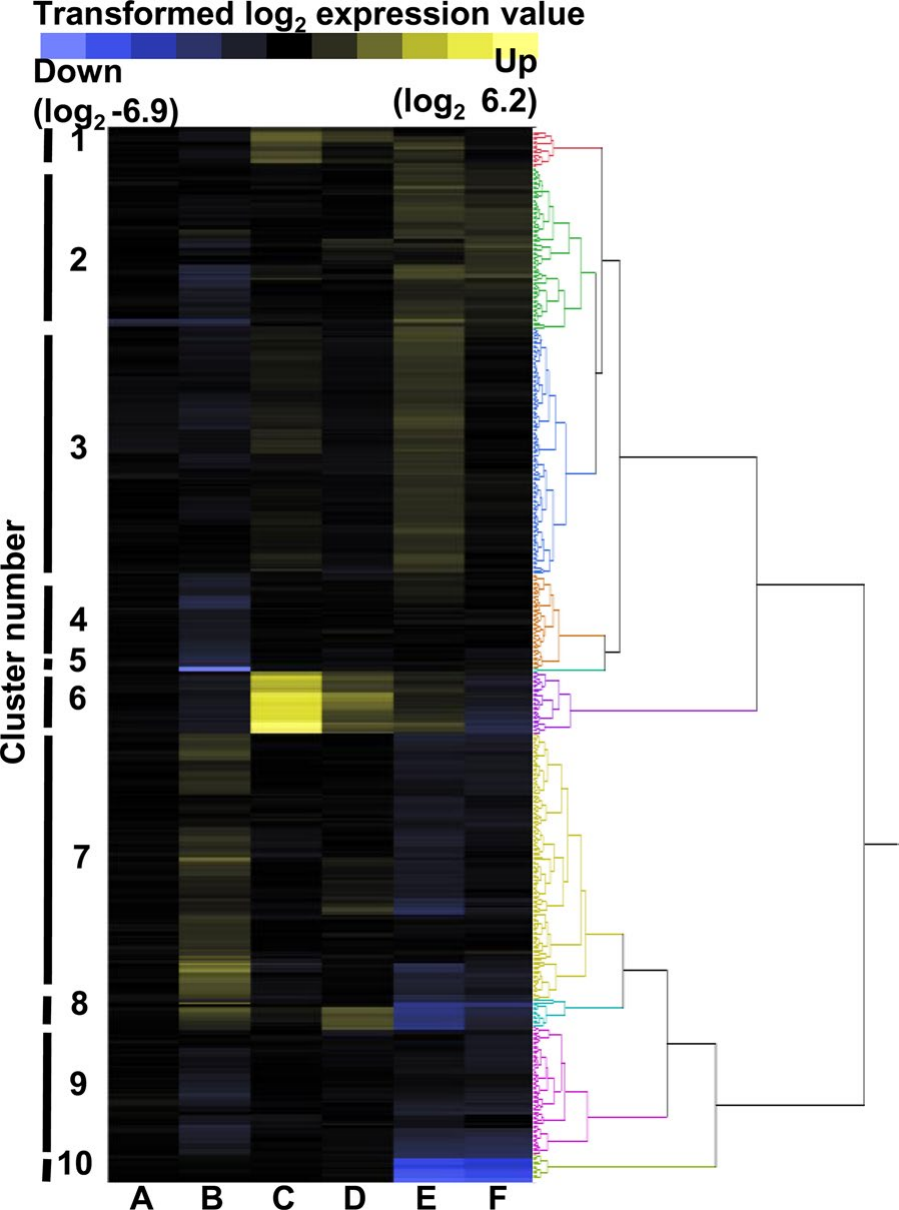
Heat map of *C. thermocellum* transcriptomic response to exogenous acetate. Hierarchical clustering of the 439 genes significantly (FDR < 0.05) differentially expressed (log_2_ ± 1) in at least one of the listed comparisons. Genes were grouped into ten clusters using the JMP Genomics 6 software. Comparisons shown *A* Wild type with acetate versus without acetate; *B ΔhydG* versus wild type, both without acetate; *C ΔhydG* with acetate versus without acetate; *D ΔhydG Δech* with acetate versus without acetate; *E ΔhydG Δech* versus *ΔhydG*, both without acetate; and *F ΔhydG Δech* versus *ΔhydG*, both with acetate. *Blue* and *yellow* indicate decreased expression and increased expression, respectively, in each comparison. Data are the average of three independent biological replicates. Cluster numbers cross-reference to Additional file 3: Data set S1

To gain insight into the cellular response to elimination of H_2_ formation and the addition of acetate, we explored changes in gene expression (Fig. 2; Additional file 3: Data set S1) and protein abundance (Additional file 4: Data set S2). A broad range of these gene expressions were also validated by RT-qPCR and a correlation coefficient of *R* ^2^ = 0.98 between microarray and qPCR analysis confirmed differential expression (Additional file 2: Fig. S3). In the absence of added acetate, 204 genes were significantly differentially expressed in *ΔhydG* compared to wild type, including 79 increased and 125 decreased in *ΔhydG* (Additional file 5: Data set S3). Genes with increased expression in *ΔhydG* included genes in Clusters 7 and 8 (Fig. 2b) associated with CRISPR (Clustered Regularly Interspaced Short Palindromic Repeats) functions (Clo1313_2705-2713; Clo1313_2970-2976), glutamate biosynthesis (Clo1313_2035-2036), and cysteine metabolism (Clo1313_2325-2331). Genes with lowered expression included genes in Cluster 6 associated with sulfate uptake and assimilation (Clo1313_0115-0125) [24], and siroheme synthesis (Clo1313_0372-0375).

Surprisingly, addition of acetate to the fermentation medium had almost no effect on the transcriptomic profile of the wild-type strain, with only three genes significantly down regulated, including one iron sulfur cluster protein and two hypothetical genes (Fig. 2a), none of which were detected by proteomics (see below). Upon addition of acetate to the growth medium of *ΔhydG*, expression of 51 genes increased and five genes decreased. The two genomic regions of Cluster 6 in particular, consisting of total 27 genes, displayed dramatic increase in expression in the presence of added acetate. Proximal genes to these genomic loci were also upregulated in the presence of acetate and grouped in Cluster 1 (Fig. 2c). The first region from Clo1313_0108-0125 includes a predicted sulfate transport system and a complete sulfate assimilatory pathway, including genes for siroheme biosynthesis, which is a cofactor for sulfite reductase. The second genomic loci Clo1313_0372-0393 also included subunits of a putative pyruvate ferredoxin oxidoreductase, a putative carbon monoxide dehydrogenase, and two ABC transporters. Addition of acetate to *ΔhydG Δech* increased expression of cluster 6 as well, though not to the same degree as *ΔhydG,* since the basal (i.e., no added acetate) level of cluster 6 was not significantly different from wild type.

The upregulation of the Clo1313_0108-0125 and Clo1313_0372-0393 gene clusters in *ΔhydG* was further confirmed by proteomics using the same cultures harvested for transcriptomic analysis. About 1700 proteins were identified, out of which 21 were significantly upregulated and 37 downregulated in *ΔhydG* upon addition of acetate. Similar to the transcriptomics, acetate addition significantly increased the abundance of proteins from two clusters in *ΔhydG* strain: Clo1313_0110-0125 increased 78—709-fold and Clo1313_0373-0391 increased 2.7—408-fold (Additional file 4: Data set S2). Taken together, these data suggest that sulfur metabolism may be perturbed in *C. thermocellum ΔhydG*.

We also considered the possibility that differential protein acetylation could account for the difference in gene expression, but no differences in acetylation were detected in the proteomics data set (Additional file 4: Data set S4). The average number of detected acetylation events per sample was comparable between wild type (172), wild type with added acetate (155), *ΔhydG* (151), and *ΔhydG* with added acetate (152). Furthermore, no individual protein had statistically significant differences in acetylation.

### Sulfur metabolism is altered in the *C. thermocellum ∆hydG* mutant

**Fig. 3:**
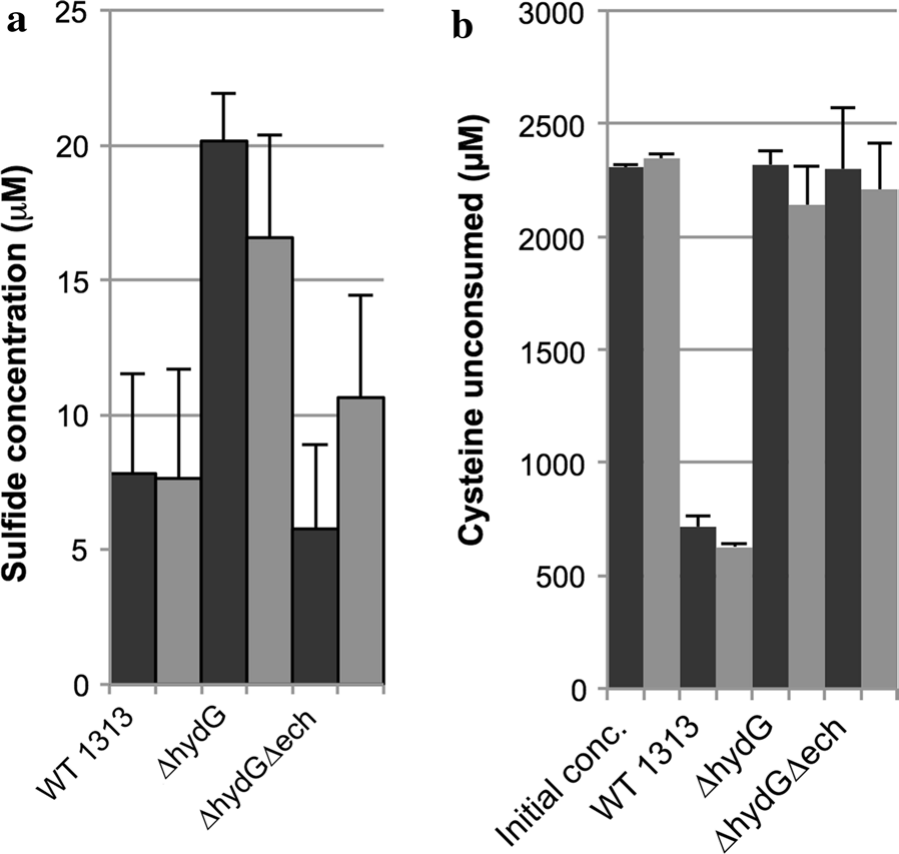
Altered sulfur flux in *C. thermocellumΔhydG* and *ΔhydG Δech*. **a** Sulfide present in fermentation broth at the end of growth. **b** Amount of cysteine present in culture supernatant after fermentation. Presence (*black bars*) and absence (*gray bars*) added acetate

With the increased abundance of transcripts and proteins related to sulfur metabolism, we examined the effect of deletion of *hydG* on sulfate reduction and cysteine metabolism. The measurement of sulfate consumption from the medium was masked by excess sulfate ions produced by the thermal degradation of the MOPS buffer during incubation at 55 °C during fermentation (Additional file 2: Fig. S3). However, sulfate is reduced to sulfide prior to incorporation into cysteine. While the wild type released ~7.5 μM sulfide into the culture medium, ~18 μM sulfide was released by *ΔhydG*, with similar results when acetate was added to the medium (Fig. 3a). As *C. thermocellum* is known to divert a significant flux of carbon and electrons toward production of secreted amino acids, we further examined the abundance of amino acids in the supernatant. Cysteine is provided in the culture medium as a reductant to make the medium anaerobic. Interestingly, the wild-type strain removed ca. 1.6 mM of 2.4 mM cysteine in the medium. *C. thermocellum ΔhydG* and *ΔhydGΔech*, on the other hand, did not consume any cysteine. Acetate addition in these mutants did not substantially alter uptake of cysteine from the medium for any of the strains (Fig. 3b).

### Genome resequencing of *C. thermocellum ΔhydG* and *ΔhydGΔech*

The improved growth of *C. thermocellum ΔhydGΔech*, even in the absence of added acetate, was surprising given that metabolism is more constrained by the deletion of the Ech hydrogenase. We, therefore, considered the possibility that additional, beneficial mutations occurred during strain construction, and that these hypothesized mutations could be influencing the phenotype. To address this, we resequenced the genomes of *C. thermocellum ΔhydG* and *ΔhydGΔech* (Additional file 1: Table S3). The parent *Δhpt* strain contained 27 mutations relative to the published genome sequence, which likely represents a combination of mutations that have accumulated during and since strain construction as well as possible errors in the published genome. The expected deletions in *hydG* and *ech* were identified in the respective strains, as was the previously identified *adhE* mutation. Nine additional mutations were identified in *ΔhydG* relative to the parent strain. In *ΔhydGΔech*, four additional mutations were identified relative to *ΔhydG*. Of these *ΔhydGΔech* mutations, two result in amino-acid changes, and both could be related to gene expression: a putative ribonuclease and a putative transcription factor.

## Discussion

Understanding the mechanisms by which growth of mutated microbes can improve is important for understanding and improving strain robustness. While the hydrogenase mutants are some of the highest ethanol yielding *C. thermocellum* strains, the slow growth phenotype would likely be prohibitive for commercialization. By combining transcriptomics, proteomics, and physiological characterization, we have shed light on a mechanism by which growth of this mutant can be improved. Furthermore, acetate is abundant in many types of pant biomass in the form of acetylated xylan. Thus, hemicellulose-derived acetate could act as a growth stimulant for this strain.

**Fig. 4:**
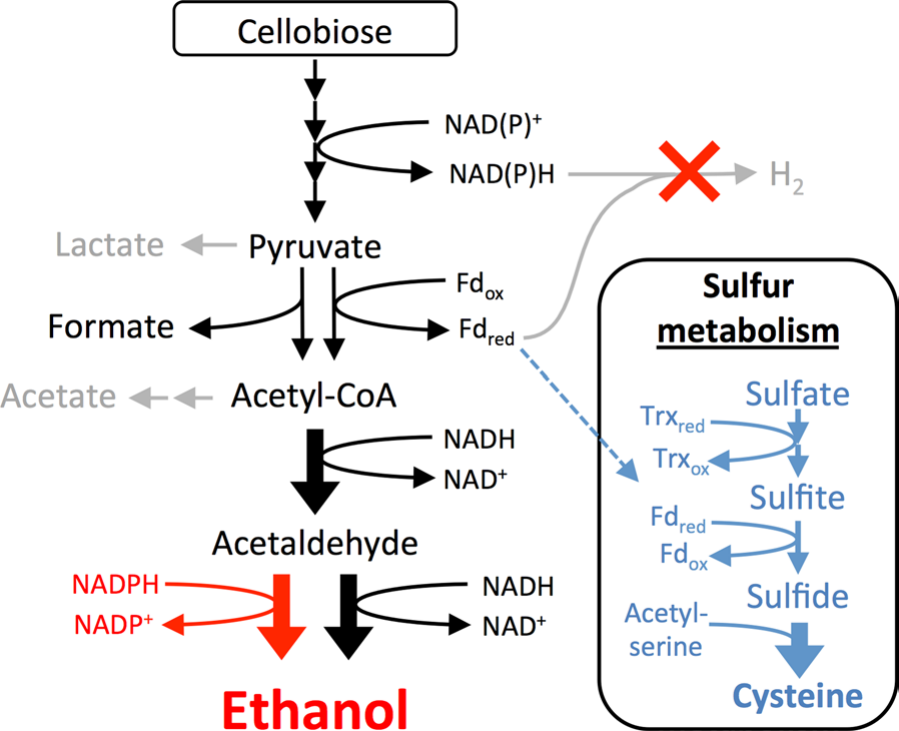
Overview of metabolic changes in *C. thermocellum ΔhydG* and *ΔhydG Δech*. When hydrogenases were inactivated (*red X*), flux to ethanol increased and sulfate reduction gene expression increased (*blue*). Furthermore, the alcohol dehydrogenase is mutated [11], allowing use of NADPH as a cofactor for ethanol production (*red pathways*). Simultaneously, flux to H_2_, lactate, and acetate decreased or was eliminated (*gray pathways*). Use of thioredoxin (Trx) and ferredoxin (Fd) as electron donors is inferred from genome annotation and has not been experimentally verified

Production of acetic acid + CO_2_ from sugars is obligately coupled to production of a more reduced compound, typically either H_2_ or formate in *C. thermocellum*, to balance redox reactions. Therefore, decreasing or eliminating H_2_ production without altering acetate production would result in a redox imbalance and decreasing flux through this pathway could help alleviate this imbalance. Thus, one might have expected that the addition of exogenous acetate could improve growth by lowering flux through the acetate production pathway. However, fermentation product profiles did not change substantially upon the addition of acetate. There was no net acetate consumption and strains are capable of growth without added acetate, demonstrating that deletion of *hydG* does not result in acetate auxotrophy. Instead, the addition of acetate to the culture medium increases the growth rate by altering sulfur metabolism (Fig. 4).

Sulfur metabolism is perturbed in *C. thermocellum ΔhydG* at the level of transcripts (Fig. 2), proteins (Additional file 4: Data set S2), and metabolites (Fig. 3). Amino-acid analysis demonstrated that *ΔhydG* strains do not consume cysteine from the medium and, therefore, must synthesize their own. One possible explanation is that deletion of *hydG* somehow blocks cysteine uptake through an unknown mechanism and thus requires the cell to make its own cysteine. Alternatively, a redox imbalance created by *ΔhydG* could be partially alleviated via sulfate reduction, which consumes four pairs of electrons per sulfate reduced to sulfide (Fig. 4). Wild type *C. thermocellum* consumed approximately 1.6 mM cysteine from the medium. If the hydrogenase mutants produce a similar amount of cysteine to fulfill cellular needs, then the necessary sulfate reduction would consume an amount of electrons equivalent to 6.4 mM H_2_ or formate. The combination of reduced acetate production relative to the wild type and increased sulfate reduction is likely sufficient to adequately balance redox reactions.

Hydrogenases are important for regenerating the oxidized ferredoxin used as the electron acceptor for Pyruvate:Ferredoxin Oxidoreductase (PFOR) during conversion of pyruvate to acetyl-CoA (Fig. 4). The sulfite reductase is predicted to utilize ferredoxin as the electron donor, which could help reoxidize the reduced ferredoxin generated by PFOR. Unlike sulfate-reducing bacteria, such as *Desulfovibrio* sp., where sulfate is used as the terminal electron acceptor in an energy-conserving electron transport chain [25, 26], in *C. thermocellum,* it is presumably an ATP-intensive pathway for cofactor re-oxidation. Although metabolically expensive, reduction of sulfate appears beneficial, as evident from the improved growth rate when this pathway is up regulated. Future efforts to provide alternate mechanisms to balance redox reactions have the potential to improve growth rate and also increase ethanol yield.

The role of the point mutation in the bifunctional alcohol/aldehyde dehydrogenase AdhE in the intersection between sulfur metabolism and redox balancing is unclear. With the expanded substrate specificity of AdhE, being able to use either NADH or NADPH for reduction of acetaldehyde to ethanol, the *ΔhydG* and *ΔhydGΔech* strains have an expanded range of possible pathways for redox balancing. However, *C. thermocellum* has a multitude of enzymes to enable the transfer of electrons between cofactors. Electrons can be directly transferred from ferredoxin to generate NADH using Rnf [27], while electrons from ferredoxin and NADH together can be used to reduce two NADP^+^ to NADPH [28, 29]. Furthermore, a carbon pathway, the malate shunt [30, 31], can functionally result in transhydrogenation during the conversion of phosphoenolpyruvate to pyruvate by oxidation of NADH by malate dehydrogenase and reduction of NADP^+^ by malic enzyme. The ability to utilize NADPH as a reductant for ethanol production would presumably provide additional flexibility in redox balancing, but the fact that sulfite reduction to sulfide is predicted to be ferredoxin-dependent makes the relevance of the mutant AdhE for sulfur metabolism tenuous.

The increased flux through sulfate reduction appears to occur both with and without added acetate, as evidenced by the lack of cysteine consumption in each case. Thus, the level of proteins involved in sulfate reduction appears to be rate limiting. The addition of acetate increases expression of the necessary genes, increasing the rate of sulfate reduction and, thus, the rate of growth. This also explains the smaller impact of acetate addition in *ΔhydGΔech*, where the expression level of this gene cluster is already at a higher basal level in the absence of added acetate. The higher basal expression presumably permits higher flux through sulfate reduction, even in the absence of added acetate. Interestingly, two non-synonymous mutations were identified in *ΔhydGΔech* in a putative ribonuclease and a transcription factor. It is conceivable that one or both of these mutations alter either mRNA stability or transcription, respectively, to return gene expression levels without added acetate to wild-type levels for gene cluster 6. Furthermore, sulfide is a volatile intermediate in the production of cysteine, so the wild-type level of sulfide production in *ΔhydGΔech* could indicate efficient assimilation of the synthesized sulfide in this strain.

Addition of acetate to wild-type cultures had a little impact, which is not surprising, because the level of added acetate (5 mM) is substantially lower than the amount wild-type strain produces during growth on 5 g/L cellobiose (approximately 9 mM under these conditions; see Table 1). However, supplementation with acetate modulates gene expression and metabolic flux in *C. thermocellum ΔhydG* by an unknown mechanism. Because of the role of hydrogenases in reoxidizing ferredoxin, deletion of *hydG* could reasonably affect flux through PFOR to perturb pools of acetyl-CoA and acetyl-phosphate, which are known to acetylate proteins to alter enzymatic properties in some organisms [32, 33]. However, no difference in protein acetylation was observed in *ΔhydG* either in the presence or absence of added acetate, suggesting that differential protein acetylation is not the likely mechanism by which sulfate reduction genes are more highly expressed. Thus, the mechanism of gene upregulation remains to be elucidated.

This transcriptomic response of *ΔhydG* to the addition of acetate is similar to that seen in wild type *C. thermocellum* upon exposure to furfural [34], a toxic aldehyde created by the dehydration of xylose at high temperature, such as during plant biomass pretreatment. In organisms like *E. coli*, furfural is detoxified via NADPH-dependent reduction to furfuryl alcohol, which is less toxic. This is thought to decrease the amount of NADPH available for biosynthesis and inhibit growth [35]. Supplementation of *E. coli* with cysteine or increasing the concentration of NADPH increased furfural tolerance, suggesting that cysteine metabolism is intimately tied to the redox state of the cell. Sulfate reduction genes are also differentially expressed in response to chemical redox perturbation [36], and addition of excess sulfate helped recover viability of a *C. thermocellum* mutant with perturbed redox metabolism [37]. How sulfur metabolism relates to redox homeostasis requires further study and may inform future metabolic engineering strategies.

## Conclusions

 Understanding and overcoming low robustness in engineered microorganisms will be essential to industrial deployment of these organisms. The previous deletion of hydrogenases in *C. thermocellum* resulted in strains with a higher ethanol yield but a substantially lower growth rate. Here, we find that this growth defect can be overcome with the addition of acetate to the medium, which results in an increase in sulfate reduction. Sulfate likely serves as an electron acceptor to help balance redox reactions, which has implications for future metabolic engineering efforts.

## Additional files

**Additional file 1: Supplemental Tables. Table S1. ** Primers used in this study. **Table S2**. Calculations of electron balance for strains with and without added acetate. **Table S3**. Analysis of mutations present in strains relative to the published wild type sequence.

**Additional file 2: Supplemental Figures. Figure S1.** Correlation plot between microarray and RT-qPCR data sets. Verification of differential gene expressions in *C. thermocellum* in the presence and absence of acetate. **Figure S2.** Concentration of amino acids produced by wild type, *ΔhydG*, and *ΔhydGΔech* strains with/out added acetate.*, asparagine quantification for two samples was prevented by interference. Error bars represent one standard deviation. **Figure S3.** Incubation of MTC medium at high temperature releases sulfate. Sulfate concentration in culture medium after incubation for 5 days. RT, room temperature; 55 °C, incubation at 55 °C; presence (black bars) and absence (gray bars) added acetate.

**Additional file 3: Data set S1.** Hierarchical clustering of gene expression. Genes significantly (p < 0.05) differentially expressed in various comparisons between the wild-type parental strain, the *ΔhydG* and *ΔhydG Δech* mutant *C. thermocellum* strains in the presence or absence of acetate. Expression differences are expressed as log2 transformed values. Yellow shading indicates upregulation by a fold change of 2 in first condition listed, while blue indicates downregulation of gene expression by a fold change of 2. For example: in the comparison, “WT (+acetate)–WT (−acetate)” yellow shading would indicate a significant upregulation of genes in the WT strain grown with acetate compared to the WT strain grown without acetate. Conversely, blue shading indicates higher expression when the cells were grown without acetate compared to those grown with acetate. Values in gray indicate the significance of the differential gene expression was below the p < 0.05 threshold for that specific comparison. Functional annotations are given of those available in the public databases. Values in the column Hierarchical cluster cross-reference to clusters in Fig. 4.

**Additional file 4: Data set S2.** Proteomic analysis of wild type and *ΔhydG* in the presence or absence of acetate. Proteomic analysis of wild type and *ΔhydG* in the presence or absence of acetate. Color code of the *p* values based on significance, with red indicating highly significant and white non-significant.

**Additional file 5: Data set S3.** Differential gene expression. Differential gene expression expressed as a ratio between the *ΔhydG*, *∆hydG ∆ech,* and wild-type strains of *C. thermocellum* in the presence or absence of acetate. Included is the False Discovery Rate (FDR) adjusted *p* value for each gene comparison with an FDR adjusted *p* value <0.05 and greater than ±1 log2 transformed ratio between the conditions indicative of altered gene regulation.

**Additional file 6: Data set S4.** Acetylation analysis of *ΔhydG* and *∆hydG∆ech* strains. Acetylation of the proteins in mutants were compared to wild type in the presence or absence of acetate.

## Abbreviations

CBP: consolidated bioprocessing
MOPS: 3-(*N*-morpholino)propanesulfonic acid
NADH: reduced nicotinamide adenine dinucleotide
NADP^+^: oxidized nicotinamide adenine dinucleotide phosphate
NADPH: reduced nicotinamide adenine dinucleotide phosphate
PFOR: pyruvate-ferredoxin oxidoreductase
SpC: spectral counts
