# Supplementary material for: Improved growth rate in Clostridium thermocellum hydrogenase mutant via perturbed sulfur metabolism
Source: Biotechnol Biofuels. 2017 Jan 3;10:6. doi: 10.1186/s13068-016-0684-x (PMC5209896; doi:10.1186/s13068-016-0684-x)
Supplement: Supplementary file 2 — Additional file 2: Supplemental Figures. Figure S1. Correlation plot between microarray and RT-qPCR data sets. Verification of differential gene expressions in C. thermocellum in the presence and absence of acetate. Figure S2. Concentration of amino acids produced by wild type, ΔhydG, and ΔhydGΔech strains with/out added acetate.*, asparagine quantification for two samples was prevented by interference. Error bars represent one standard deviation. Figure S3. Incubation of MTC medium at high temperature releases sulfate. Sulfate concentration in culture medium after incubation for 5 days. RT, room temperature; 55 °C, incubation at 55 °C; presence (black bars) and absence (gray bars) added acetate. [file 13068_2016_684_MOESM2_ESM.docx]

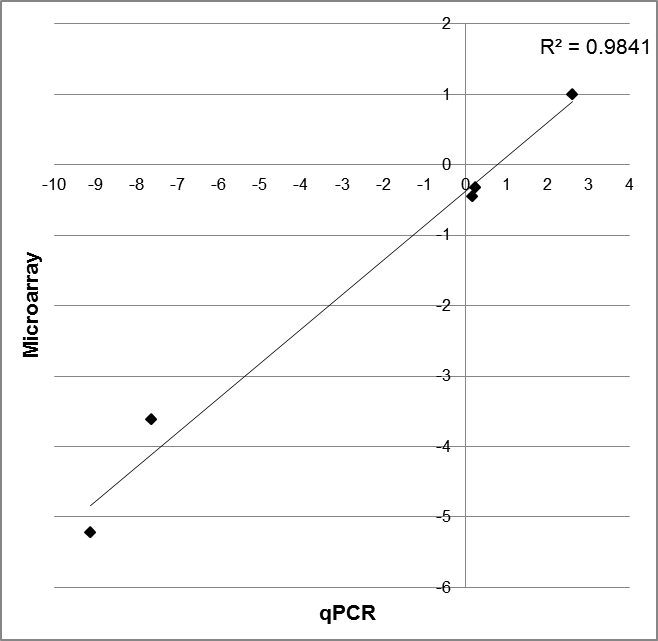


**Supplementary Fig S1. Correlation plot between microarray and RT-qPCR datasets**. Verification of differential gene expressions in C. thermocellum in the presence and absence of acetate.

**Supplementary Fig. S2**: Concentration of amino acids produced by wild-type, ΔhydG, and ΔhydGΔech strains with/out added acetate.*, asparagine quantification for two samples was prevented by interference. Error bars represent one standard deviation.


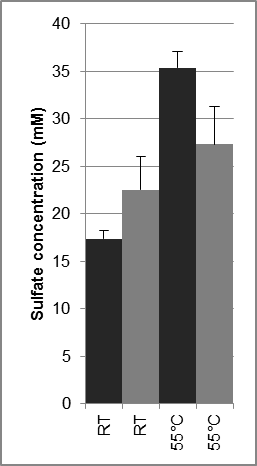


**Supplementary Fig S3. Incubation of MTC medium at high temperature releases sulfate**. Sulfate concentration in culture medium after incubation for 5 days. RT, room temperature; 55°C, incubation at 55°C; presence (black bars) and absence (gray bars) added acetate.
